# Bioinformatics Studies Provide Insight into Possible Target and Mechanisms of Action of Nobiletin against Cancer Stem Cells

**DOI:** 10.31557/APJCP.2020.21.3.611

**Published:** 2020-03

**Authors:** Adam Hermawan, Herwandhani Putri

**Affiliations:** 1 *Department of Pharmaceutical Chemistry, *; 2 *Cancer Chemoprevention Research Center, Faculty of Pharmacy, Universitas Gadjah Mada, Yogyakarta, Indonesia. *

**Keywords:** Nobiletin, anticancer, bioinformatics, cancer stem cells, signaling pathway

## Abstract

**Objective:**

Nobiletin treatment on MDA-MB 231 cells reduces the expression of *CXC* chemokine receptor type 4 (CXCR4), which is highly expressed in cancer stem cell populations in tumor patients. However, the mechanisms of nobiletin in cancer stem cells (CSCs) remain elusive. This study was aimed to explore the potential target and mechanisms of nobiletin in cancer stem cells using bioinformatics approaches.

**Methods:**

Gene expression profiles by public COMPARE predicting the sensitivity of tumor cells to nobiletin. Functional annotations on gene lists are carried out with The Database for Annotation, Visualization and Integrated Discovery (DAVID) v6.8, and WEB-based GEne SeT Analysis Toolkit (WebGestalt). The protein-protein interaction (PPI) network was analyzed by STRING-DB and visualized by Cytoscape.

**Results:**

Microarray analyses reveal many genes involved in protein binding, transcriptional and translational activity. Pathway enrichment analysis revealed breast cancer regulation of estrogen signaling and Wnt/ß-catenin by nobiletin. Moreover, three hub genes, i.e. *ESR1*, *NCOA3*, and *RPS6KB1* and one significant module were filtered out and selected from the PPI network.

**Conclusion:**

Nobiletin might serve as a lead compound for the development of CSCs-targeted drugs by targeting estrogen and Wnt/ß-catenin signaling. Further studies are needed to explore the full therapeutic potential of nobiletin in cancer stem cells.

## Introduction

Recent studies have shown that the ability of tumors to develop and propagate depends on a small population of cells called cancer stem cells (CSCs) (Pan et al., 2018; Zhu and Fan, 2018). CSCs are responsible for resistance to chemotherapy and radiotherapy (Toledo-Guzman et al., 2018). Conventional chemotherapy has proved to be able to reduce tumor size; however most of the tumor relapsed because the population of CSCs that were able to survive and grow into tumor bulk (Zhu and Fan, 2018). The CSC-targeted therapy will target the CSCs population whose slowly growth (Moltzahn et al., 2008), and thus the effectiveness of cancer therapy will be achieved. Collectively, CSC-targeted therapy is needed to prevent relapse after chemotherapy. 

**Figure 1:**
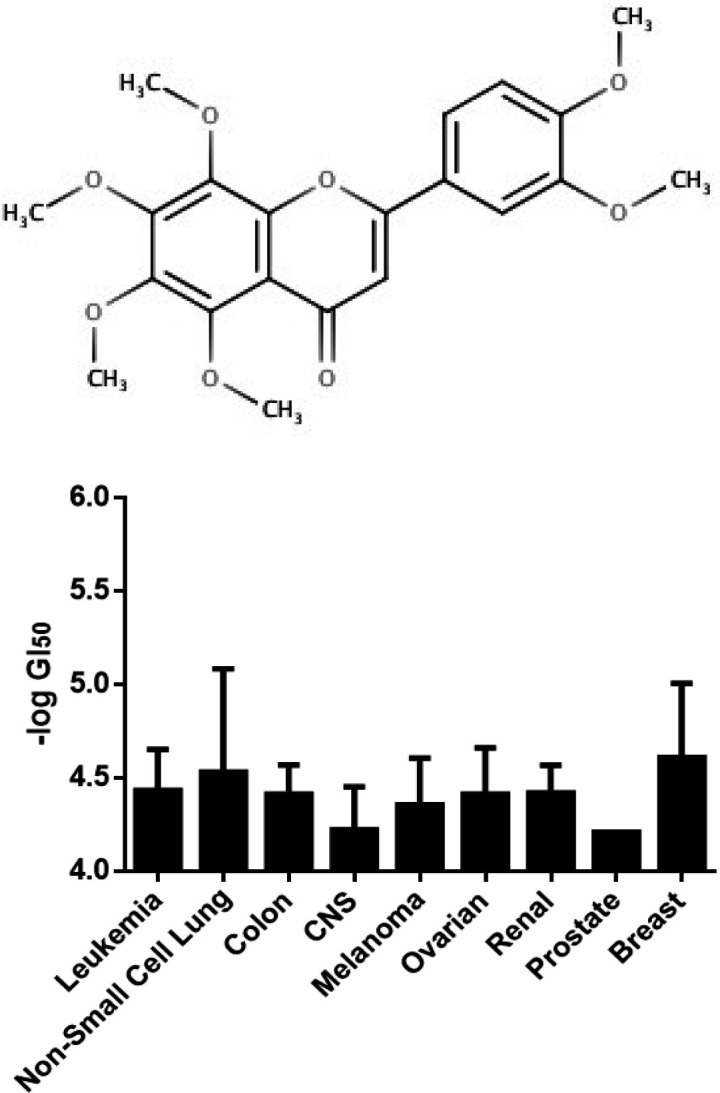
(A) Chemical Structure of Nobiletin. (B) Cytotoxicity of Nobiletin on the NCI-60 Tumor Cell Line Panel

Flavonoid compounds have been shown to overcome chemoresistance (Meiyanto et al., 2012) and to inhibit CSCs (Hermawan and Putri, 2018). One potential flavonoid compound to be developed as CSC-targeted drugs is nobiletin (Figure 1A). Previous studies showed that polymetoxiflavone citrus flavonoids namely nobiletin exhibits cytotoxic effects on several cancer cells, e.g. TMK-1, MKN-45, MKN-74 and KATO-III stomach cancer cells (Yoshimizu et al., 2004), MH1C1 and HepG2 human hepatocellular carcinoma (Ohnishi et al., 2004), MDA-MB-435 breast cancer cells, MCF-7 and in HT-29 colon cancer cells (Morley et al., 2007). Studies on the combination of nobiletin and conventional chemotherapy agents have also been carried out. Nobiletin is reported to increase the uptake of chemotherapy vinblastine through inhibition of P-gp in Caco-2 cells (Takanaga et al., 2000). Nobiletin also increased doxorubicin cytotoxicity in MCF-7 breast cancer cells but not T47D cells (Meiyanto et al., 2011). In addition, nobiletin showed the effect of inhibiting metastasis by downregulating CXC chemokine receptor type 4 (CXCR4) and matrix metallopeptidase-9 on MDA-MB 231 breast cancer cells (Baek et al., 2012). Therefore, it has been proven that nobiletin is able to overcome chemoresistance and also inhibit CXCR4 which is one of the regulators of CSCs, but its molecular mechanism on CSCs need to be clarified further.

In this study, we used comprehensive bioinformatics analysis to explore nobiletin cytotoxicity and mechanism in CSCs. Analysis of the public library from the COMPARE database was done to produce a list of drugs that have similarities with nobiletin, as well as a gene list that was influenced by nobiletin on the NCI 60 cell line panel. From the microarray data, functional annotations are then carried out to predict molecular mechanisms, functions and roles of these genes. Furthermore, an analysis of protein-protein interaction was performed from the gene list. Hence we provide information about the possible molecular mechanisms of the nobiletin and its molecular targets against cancer stem cells.

## Materials and Methods

*Data collection and processing*

Cytotoxicity and mRNA arrays data were obtained from the NCI 60 DTP website (http.dtp.nci.nih.gov) (Monks et al., 1997). COMPARE analysis with the public library produces a list of drugs that have similarities with nobiletin, as well as a list of gene expressions on the NCI 60 cell line panel (Mahmoud et al., 2018). The similarity pattern is expressed as the Pearson correlation coefficient. In this study, the list of compounds and genes was limited to the Pearson correlation coefficient <-0.5 and> 0.5.

*Functional and pathway enrichment analysis*

Gene ontology (GO) and Kyoto Encyclopedia of Genes and Genomes (KEGG) pathway enrichment analysis were carried out by The Database for Annotation, Visualization and Integrated Discovery (DAVID) v6.8 (Huang da et al., 2009), with p<0.05 was selected as the cutoff value. Moreover, pathway enrichment was also conducted busing Overrepresentation Enrichment Analysis (ORA) from WEB-based GEne SeT AnaLysis Toolkit (WebGestalt) with FDR<0.05 was selected as the cutoff value (Wang et al., 2017a). 

*Construction of PPI network and module analysis*

Protein-protein interaction (PPI) network was constructed with STRING-DB v11.0 (Szklarczyk et al., 2015). Confidence scores >0.7 were considered significant. PPI network was visualized by Cytoscape software. Genes with a degree score more than 5, analyzed by CytoHubba plugin, were selected as hub genes.

**Table 1:** Correlation of Nobiletin to Standard Agent by COMPARE Analyses with Log IC_50_ of Nobiletin

No	Correlation coefficient	NSC Code	Drugs
1	0.525	S180973	Tamoxifen
2	0.501	S280594	Triciribine Phosphate
3	0.453	S349438	4-ipomeanol
4	0.386	S95580	Hexamethylenebisace Tamide
5	0.339	S180973	Tamoxifen
6	0.331	S118994	Diglycoaldehyde
7	0.327	S73754	Fluorodopan
8	0.31	S51143	Impy
9	0.309	S349156	Pancratiastatin
10	0.307	S141540	VP-16 (Etoposide)
11	0.301	S357704	Cyanomorpholino- ADR

**Table 2:** mRNA Expression Analysed by COMPARE with log IC_50_ of Nobiletin on the NCI-60 Cell Line Panel

No	Pearson Correlation Coefficient	Gene Symbol	Gene Name
1	0.612	*FRAT2 *	Frequently Rearranged In Advanced T-Cell Lymphomas 2
2	0.586	*AANAT *	Aralkylamine N-Acetyltransferase
3	0.582	*DYM *	Dymeclin
4	0.576	*SNHG8 *	Small Nucleolar RNA Host Gene 8
5	0.574	*LETMD1 *	LETM1 Domain Containing 1
6	0.571	*ATXN7L3B *	Ataxin 7 Like 3B
7	0.559	*EPB41L5 *	Erythrocyte Membrane Protein Band 4.1 Like 5
8	0.557	*PISD *	Phosphatidylserine Decarboxylase
9	0.557	*ALDH3B2 *	Aldehyde Dehydrogenase 3 Family Member B2
10	0.555	*VPS37C *	VPS37C, ESCRT-I Subunit
11	0.555	*HEATR6 *	HEAT Repeat Containing 6
12	0.554	*LARP4B *	La Ribonucleoprotein Domain Family Member 4B
13	0.554	*FBP1 *	Fructose-Bisphosphatase 1
14	0.554	*AIF1L *	Allograft Inflammatory Factor 1 Like
15	0.55	*SMARCD2*	SWI/SNF Related, Matrix Associated, Actin Dependent Regulator of Chromatin, Subfamily D, Member 2
16	0.548	*GRTP1 *	Growth Hormone Regulated TBC Protein 1
17	0.546	*C21orf33*	Chromosome 21 Open Reading Frame 33
18	0.545	*WDR25 *	WD Repeat Domain 25
19	0.544	*TEAD2 *	TEA Domain Transcription Factor 2
20	0.544	*EIF4B *	Eukaryotic Translation Initiation Factor 4B
21	0.543	*FRAT1 *	Frequently Rearranged In Advanced T-Cell Lymphomas 1
22	0.542	*TREH *	Trehalase
23	0.542	*NCOA3 *	Nuclear Receptor Coactivator 3
24	0.541	*RMND1 *	Required for Meiotic Nuclear Division 1 Homolog
25	0.541	*ALOX15 *	Arachidonate 15-Lipoxygenase
26	0.54	*TRIM37 *	Tripartite Motif Containing 37
27	0.538	*TMEM241 *	Transmembrane Protein 241
28	0.538	*APRT *	Adenine Phosphoribosyltransferase
29	0.537	*SP1 *	Sp1 Transcription Factor
30	0.536	*USP32*	Ubiquitin Specific Peptidase 32
31	0.535	*RNF44 *	Ring Finger Protein 44
32	0.535	*BRIP1 *	BRCA1 Interacting Protein C-Terminal Helicase 1
33	0.534	*RLN2 *	Relaxin 2
34	0.534	*NPY1R *	Neuropeptide Y Receptor Y1
35	0.534	*ITGA2B *	Integrin Subunit Alpha 2b
36	0.533	*ZNF282 *	Zinc Finger Protein 282
37	0.533	*RHPN1 *	Rhophilin Rho Gtpase Binding Protein 1
38	0.533	*MEPCE *	Methylphosphate Capping Enzyme
39	0.532	*SPTSSB *	Serine Palmitoyltransferase Small Subunit B
40	0.53	*SPDEF *	SAM Pointed Domain Containing ETS Transcription Factor
41	0.53	*PIK3R2 *	Phosphoinositide-3-Kinase Regulatory Subunit 2
42	0.53	*EIF3E *	Eukaryotic Translation Initiation Factor 3 Subunit E
43	0.529	*NUP210L*	Nucleoporin 210 Like
44	0.528	*ELP2 *	Elongator Acetyltransferase Complex Subunit 2
45	0.527	*GATA3 *	GATA Binding Protein 3
46	0.526	*PPM1D *	Protein Phosphatase, Mg2+/Mn2+ Dependent 1D
47	0.526	*IRX5 *	Iroquois Homeobox 5
48	0.525	*TFF1*	Trefoil Factor 1
No	Pearson Correlation Coefficient	*Gene Symbol*	Gene Name
49	0.525	*RAD51C *	RAD51 Paralog C
50	0.525	*ESR1 *	Estrogen Receptor 1
51	0.524	*RFX1 *	Regulatory Factor X1
52	0.524	*C15orf59 *	Chromosome 15 Open Reading Frame 59
53	0.523	*ZNF277 *	Zinc Finger Protein 277
54	0.523	*PABPC1 *	Poly(A) Binding Protein Cytoplasmic 1
55	0.523	*CYB561*	Cytochrome B561
56	0.522	*SCAMP1 *	Secretory Carrier Membrane Protein 1
57	0.522	*PLEKHF2*	Pleckstrin Homology And FYVE Domain Containing 2
58	0.522	*KIAA1324 *	Kiaa1324
59	0.522	*DSCAM *	DS Cell Adhesion Molecule
60	0.521	*XBP1 *	X-Box Binding Protein 1
61	0.521	*TUBD1 *	Tubulin Delta 1
62	0.52	*EMCN *	Endomucin
63	0.52	*APPBP2 *	Amyloid Beta Precursor Protein Binding Protein 2
64	0.519	*TMEM18 *	Transmembrane Protein 18
65	0.519	*ST6GALNAC4 *	ST6 N-Acetylgalactosaminide Alpha-2,6-Sialyltransferase 4
66	0.519	*SPPL2B *	Signal Peptide Peptidase Like 2B
67	0.518	*TMEM183A *	Transmembrane Protein 183A
68	0.517	*PSMD6 *	Proteasome 26S Subunit, Non-Atpase 6
69	0.517	*ECSIT *	ECSIT Signalling Integrator
70	0.516	*SIAH2 *	Siah E3 Ubiquitin Protein Ligase 2
71	0.516	*POU6F2-AS2 *	POU6F2 Antisense RNA 2
72	0.516	*MAX*	MYC Associated Factor X
73	0.516	*GNAO1 *	G Protein Subunit Alpha O1
74	0.515	*SPATA17*	Spermatogenesis Associated 17
75	0.514	*STARD10*	Star Related Lipid Transfer Domain Containing 10
76	0.513	*PATZ1 *	POZ/BTB And AT Hook Containing Zinc Finger 1
77	0.512	*PDZD3 *	PDZ Domain Containing 3
78	0.512	*CYP2J2 *	Cytochrome P450 Family 2 Subfamily J Member 2
79	0.512	*COX6C *	Cytochrome C Oxidase Subunit 6C
80	0.511	*PLXNA4*	Plexin A4
81	0.511	*PCDHB4*	Protocadherin Beta 4
82	0.51	*TBC1D30 *	TBC1 Domain Family Member 30
83	0.51	*PREX1 *	Phosphatidylinositol-3,4,5-Trisphosphate Dependent Rac Exchange Factor 1
84	0.51	*MKS1 *	Meckel Syndrome, Type 1
85	0.509	*ZNF768 *	Zinc Finger Protein 768
86	0.509	*PARD6B *	Par-6 Family Cell Polarity Regulator Beta
87	0.509	*PABPC3 *	Poly(A) Binding Protein Cytoplasmic 3
88	0.509	*GATC *	Glutamyl-Trna Amidotransferase Subunit C
89	0.508	*TRPC5OS *	TRPC5 Opposite Strand
90	0.508	*KREMEN2 *	Kringle Containing Transmembrane Protein 2
91	0.508	*HOOK2*	Hook Microtubule Tethering Protein 2
92	0.507	*RPS6KB1*	Ribosomal Protein S6 Kinase B1
93	0.507	*CEACAM21 *	Carcinoembryonic Antigen Related Cell Adhesion Molecule 21
94	0.507	*ABCA12 *	ATP Binding Cassette Subfamily A Member 12
95	0.506	*MVK *	Mevalonate Kinase
96	0.505	*DHTKD1 *	Dehydrogenase E1 And Transketolase Domain Containing 1
No	Pearson Correlation Coefficient	*Gene Symbol*	Gene Name
97	0.504	*TDRD5*	Tudor Domain Containing 5
98	0.504	*ENTHD2 *	Tepsin
99	0.503	*SLC29A2 *	Solute Carrier Family 29 Member 2
100	0.501	*MRPL4 *	Mitochondrial Ribosomal Protein L4
101	0.501	*MATK *	Megakaryocyte-Associated Tyrosine Kinase
102	0.501	*FBXW9 *	F-Box And WD Repeat Domain Containing 9
103	0.501	*EIF3C *	Eukaryotic Translation Initiation Factor 3 Subunit C
104	0.5	*RXRA *	Retinoid X Receptor Alpha
105	-0.505	*BLOC1S6 *	Biogenesis Of Lysosomal Organelles Complex 1 Subunit 6
106	-0.51	*NR2F6 *	Nuclear Receptor Subfamily 2 Group F Member 6
107	-0.514	*VANGL1 *	VANGL Planar Cell Polarity Protein 1
108	-0.564	*SEPT2*	Septin 2

Positive correlation coefficients indicate direct correlation to logIC_50_ value whereas negative correlation coeficients showed inverse correlation.

**Table 3:** The Top Five Gene Ontology and KEGG Pathway Enrichment of DEGs, Analysed by DAVID

ID	Term	Count	P value	Genes
Biological Process				
GO:0001731	Formation of translation preinitiation complex	3	0.006970337	*EIF3C, EIF4B, EIF3E*
GO:0032869	Cellular response to insulin stimulus	4	0.008691289	*SP1, XBP1, APRT, PIK3R2*
GO:0014065	Phosphatidylinositol 3-kinase signaling	3	0.009534075	*XBP1, GATA3, PIK3R2*
GO:0006446	Regulation of translational initiation	3	0.016576226	*EIF3C, EIF4B, EIF3E*
GO:0009267	Cellular response to starvation	3	0.027361724	*MAX, PPM1D, KIAA1324*
Cellular component				
GO:0000790	Nuclear chromatin	6	0.003712494	*SP1, NCOA3, SMARCD2, RXRA, GATA3, ESR1*
GO:0005829	Cytosol	29	0.006647911	*RHPN1, PREX1, STARD10, RPS6KB1, BLOC1S6, EIF3C, HOOK2, XBP1, EIF3E, FRAT1, AANAT, FRAT2, PABPC1, DHTKD1, PSMD6, PIK3R2, ABCA12, MATK, PARD6B, FBP1, LARP4B, APRT, EIF4B, TRIM37, MKS1, ALOX15, MVK, SIAH2, PDZD3*
GO:0005654	Nucleoplasm	25	0.009918189	*RAD51C, RPS6KB1, BLOC1S6, MAX, SMARCD2, XBP1, EIF3E, GATA3, NR2F6, PATZ1, PSMD6, SCAMP1, RXRA, ESR1, SPPL2B, BRIP1, TEAD2, ECSIT, APRT, RNF44, NCOA3, SP1, TUBD1, RFX1, SIAH2*
Molecular function				
GO:0005515	Protein binding	62	0.007869723	*RAD51C, SEPT2, PREX1, RPS6KB1, VPS37C, HOOK2, MAX, SMARCD2, GATA3, NR2F6, FRAT1, PSMD6, RMND1, DSCAM, MATK, SCAMP1, MRPL4, VANGL1, RXRA, ESR1, FBP1, ECSIT, TRIM37, MKS1, PPM1D, ALOX15, NCOA3, MVK, SIAH2, ITGA2B, GATC, RHPN1, STARD10, EIF3C, BLOC1S6, FBXW9, XBP1, EIF3E, AANAT, LETMD1, TFF1, PABPC1, APPBP2, TMEM183A, USP32, ABCA12, PIK3R2, PARD6B, SPTSSB, SPPL2B, BRIP1, TEAD2, NPY1R, LARP4B, CYB561, EIF4B, PLEKHF2, SP1, WDR25, RFX1, DYM, PDZD3*
GO:0004879	RNA polymerase II transcription factor activity, ligand-activated sequence-specific DNA binding	3	0.017092174	*RXRA, NR2F6, ESR1*
GO:0001046	Core promoter sequence-specific DNA binding	3	0.02389184	*SP1, GATA3, ESR1*
GO:0043565	Sequence-specific DNA binding	8	0.025500996	*MAX, IRX5, SP1, XBP1, RXRA, NR2F6, SPDEF, ESR1*
GO:0003707	Steroid hormone receptor activity	3	0.038898918	*RXRA, NR2F6, ESR1*
KEGG pathway enrichment analysis				
hsa03013	RNA transport	6	0.005016166	*EIF3C, EIF4B, EIF3E, PABPC3, PABPC1, NUP210L*
hsa05222	Small cell lung cancer	4	0.017791047	*MAX, RXRA, ITGA2B, PIK3R2*
hsa04915	Estrogen signaling pathway	4	0.026525517	*GNAO1, SP1, ESR1, PIK3R2*
hsa04919	Thyroid hormone signaling pathway	4	0.038861334	*NCOA3, RXRA, ESR1, PIK3R2*
hsa04150	mTOR signaling pathway	3	0.054897616*	*EIF4B, RPS6KB1, PIK3R2*
hsa03013	RNA transport	6	0.005016166	*EIF3C, EIF4B, EIF3E, PABPC3, PABPC1, NUP210L*

*, not significant

**Figure 2.**
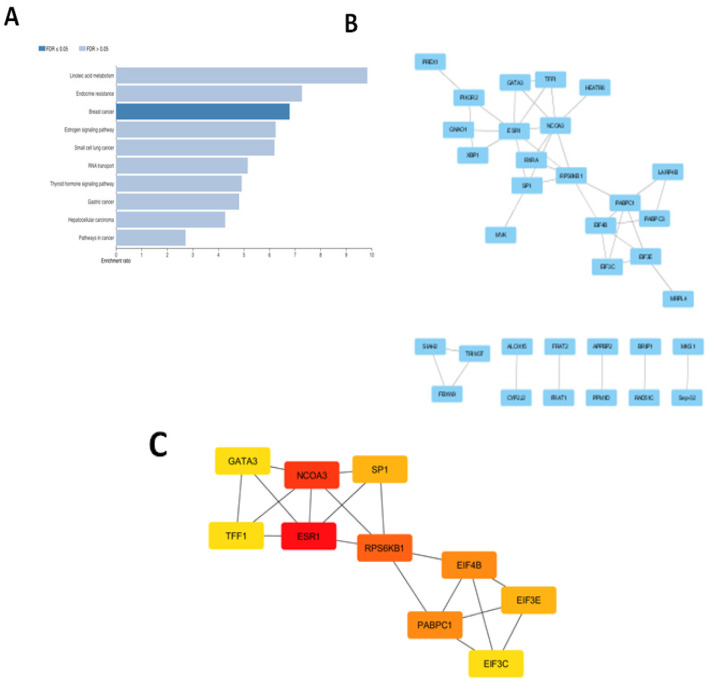
(A), Pathway enrichment analysis of DEGs with Webgestalt; (B), Protein-protein interaction networks of DEGs, analyzed with STRING-DB and Cytoscape; (C), Top 10 hub genes with the highest degree score, analyzed by Cytoscape

**Table 4:** DEGs Involved in Breast Cancer Regulation, Pathway Enrichment Analysis by WebGestalt

User ID	Gene Symbol	Gene Name
RPS6KB1	*RPS6KB1*	ribosomal protein S6 kinase B1
ESR1	*ESR1*	estrogen receptor 1
PIK3R2	*PIK3R2*	phosphoinositide-3-kinase regulatory subunit 2
SP1	*SP1*	Sp1 transcription factor
NCOA3	*NCOA3*	nuclear receptor coactivator 3
FRAT1	*FRAT1*	FRAT1, WNT signaling pathway regulator
FRAT2	*FRAT2*	FRAT2, WNT signaling pathway regulator

**Table 5:** The Hub Genes Identified by PPI Networks, Possessing Degree more than 5

Gene Symbol	Gene name	Degree score
*ESR1*	estrogen receptor 1	8
*NCOA3*	nuclear receptor coactivator 3	7
*RPS6KB1*	ribosomal protein S6 kinase B1	6

## Results

*COMPARE analysis reveals mRNA target list and standard agent*

This study explored the molecular mechanism of nobiletin in CSCs. Analysis of cytotoxicity with a public database of COMPARE showed that nobiletin exhibits cytotoxicity at the same level in the NCI-60 cells panel showing by similar IC_50_ value (Figure 1B). COMPARE analysis identified 11 standard agents which have a correlation with nobiletin (Table 1). Tamoxifen, triciribine phosphate and 4-ipomeanol are standard drugs with the highest score of a Pearson correlation coefficient. 

Level of mRNA expression analysed by COMPARE showed 108 genes regulated by nobiletin (Table 2), which 104 and 4 genes with positive and negative Pearson correlation coefficient, respectively. *FRAT2*, *AANAT*, *DYM* and *SNHG8* are genes with direct correlation, whereas *BLOC1S6,* *NR2F6*, *VANGL1* and *SEPT2* are genes with inverse correlation. Direct correlation indicates that the higher mRNA expression, the higher the chemoresistance, while inverse correlation indicates that the higher mRNA expression, the higher the chemosensitivity. *FRAT2 *has the highest Pearson correlation coefficient (0.612) while *FRAT1* shows the Pearson correlation coefficient of 0.543. Both *FRAT1* and *FRAT2* are regulatory genes of Wnt/ß-catenin signaling. *ESR1* shows the Pearson correlation coefficient of 0.525. 

*Gene ontology analysis of potential nobiletin target genes*

Gene ontology analysis was classified into biological process, cellular component and molecular function (Table 3). There are no significant GO analysis results of the genes with a negative Pearson correlation coefficient. We found that the upregulated genes mostly involved in the biological process related to negative regulation of transcription, translational initiation, phosphatidylinositol 3-kinase signaling and cellular response to insulin stimulus. Moreover, the upregulated genes located in the cellular component of nuclear chromatin, cytosol and cytoplasm (e.g. *ESR1* and *NCOA3*), and play a role in the molecular function of protein binding, transcriptional and translational activity, as well as steroid hormone activity, e.g. ESR1. 

*KEGG pathway enrichment, protein-protein interaction (PPI) network construction and module selection*

KEGG pathway enrichment indicated several pathways regulated by nobiletin (Table 3) such as RNA transport, small cell lung cancer, estrogen signaling pathway and thyroid hormone signaling pathway. Pathway enrichment analysed by WebGestalt showed breast cancer signaling regulated by nobiletin (Figure 2A). In addition, several genes involved in breast cancer regulation by targeting estrogen receptor and Wnt/ß-catenin signaling (Table 4). A total of 108 genes were constructed to PPI network complex containing 105 nodes and 40 edges, with average node degree 0.762 (Figure 2B). Three nodes with a degree score more than five were identified as hub genes, e.g. *ESR1*, *NCOA3* and *RPS6KB1* (Figure 2C and Table 5).

## Discussion

This study analyzed the molecular mechanism of nobiletin in CSCs using bioinformatics approaches. A pharmacological network analysis using bioinformatics approach can help to explain the potential target and mechanism of compounds in several diseases (Lee et al., 2018). Analysis of cytotoxicity with a public database of COMPARE showed that nobiletin exhibits cytotoxicity at the same level in NCI-60 cells panel showing by similar IC50 value. Nobiletin cytotoxicity does not depend on particular tissue. The low IC_50_ value indicates the potential of nobiletin for CSCs-targeted agents in combinatorial chemotherapy. The ideal compounds for combinatorial therapy should be potent, have low toxicity and selective (Wang et al., 2014).

COMPARE analysis identified 11 standard agents which have a correlation with nobiletin (Table 1). Tamoxifen, triciribine phosphate and 4-ipomeanol are standard drugs with the highest score of a Pearson correlation coefficient. Tamoxifen is a classical selective estrogen receptor modulator (SERM) for adjuvant chemotherapy of estrogen receptor-positive (Daurio et al., 2016). Tamoxifen activates tumor suppressor gene maspin in breast cancer (Liu et al., 2004). 4-ipomeanol, a lung-toxic furanoterpenoid produced by sweet potatoes (Ipomoea batatas) infected with the fungus Fusarium solani (Boyd and Wilson, 1972; Lakhanpal et al., 2001), is the first agent to undergo preclinical study at the National Cancer Institute (NCI) based on a specific biochemical-biological rationale for clinical investigation as an antineoplastic agent targeted lung cancer (Christian et al., 1989). Phase I and phase II clinical trial of 4-ipomeanol in patients with non-small cell lung cancer and advanced hepatocellular carcinoma, respectively showed that 4-ipomeanol is not recommended for those diseases (Kasturi et al., 1998; Lakhanpal et al., 2001). Triciribine, an inhibitor of Akt phosphorylation and activation, reduces CSC population in T-cell acute lymphoblastic leukemia cells (Evangelisti et al., 2011) and human breast cancer cells SKBR3 cells (Jain et al., 2015). Accordingly, nobiletin probably acts as a kinase inhibitor in inhibiting CSCs. 

COMPARE analysis showed that *FRAT1* and* FRAT2 *are genes with positive, while *VANGL1* is genes with negative Pearson correlation coefficient, respectively. Those genes also involve in the Wnt/ß-catenin signaling pathway. The frequently rearranged in advanced T-cell lymphomas 1 (Frat 1) and 2 (Frat 2) are positively regulator of the Wnt signaling pathway by stabilizing ß-catenin through the association with GSK-3 (Saitoh et al., 2001). Upon binding to GSK3, Frat prevents the phosphorylation and accompanying degradation of ß-catenin and allows the activation of downstream target genes (van Amerongen and Berns, 2005; Luan et al., 2008). Wnt/ß-catenin signalling may be aberrantly activated through Frat1 overexpression in ovarian serous adenocarcinomas (Wang et al., 2006). The expression of Frat is also positively correlated with the degree of tumor differentiation and the abnormal cell expression of ß-catenin in lung cancer (Luan et al., 2008). Overexpression of Frat1 and abnormal expression of β-catenin were found to represent a poor prognosis for the non-small cell lung cancer patients (Zhang et al., 2012). Frat1 demonstrates oncogenic properties in prostate cancer by inhibiting GSK 3β against β-catenin and thus promoting cell growth (Zhang et al., 2016), while Frat2 mediates the oncogenic activation of Rac by mixed lineage leukemia fusions (Walf-Vorderwulbecke et al., 2012). *VANGL1* encodes a transmembrane protein that interacts with Frizzeld a receptor of Wnt (Jenny et al., 2003) and negatively regulates canonical Wnt/β-catenin signaling in mammalian cells. *FRAT1* and* FRAT2* are tumor promoting genes whereas* VANGL1* is a tumor suppressor gene which involved in the Wnt/ß-catenin signaling pathway and thus posses as a molecular target of nobiletin. 

KEGG pathway enrichment analysis revealed that estrogen and Wnt/ß-catenin signaling are regulated by nobiletin. There is only a few studies on the role of estrogen in BCSCs. A study demonstrated that estrogen treatment reduces mammosphere formation from estrogen receptor-positive breast cancer cells (Simoes et al., 2011). Other studies showed that estrogen signaling blocking by tamoxifen induces chemoresistance due to EGFR and estrogen receptor cross talk (Shou et al., 2004). Expression of Wnt/ß-catenin signaling pathway-regulated genes correlates with estrogen receptor expression (Lamb et al., 2013). Activation of Wnt/ß-catenin signaling and CSCs properties are associated with advanced progression of ER-positive breast cancer (Sun et al., 2018). The Wnt/ß-catenin pathway is considered to be one of the most important pathways in the regulation of CSCs (Wang et al., 2016). A study showed that Wnt/ß-catenin and estrogen signaling pathways cross-talk in vivo through functional interaction between ERα and β-catenin (Kouzmenko et al., 2004). Therefore it is interesting to further explore the effect of nobiletin in estrogen and Wnt/ß-catenin signaling as well as its cross-talk in CSCs. 

Pathway enrichment analysis with KEGG also showed the mTOR pathway regulated by nobiletin even the p-value is slightly greater than the cut off (p= 0.0548). The PI3K/Akt/mTOR signaling pathway is important for CSCs maintenance and could be a promising target for development of CSC-target drugs (Matsubara et al., 2013; Xia and Xu, 2015; Dandawate et al., 2016; Francipane and Lagasse, 2016). Rapamycin and triciribine target CSC population, and inhibits migration and invasion on glioblastoma and neuroblastoma cells (Bahmad et al., 2018). COMPARE analysis showed that triciribin is one of the compounds with the highest similarity to nobiletin, and therefore the effect of nobiletin in mTOR signaling is also potential to be further explored.

There are three hub genes identified from PPI networks, i.e. *ESR1*, *NCOA3*, *RPS6KB1 ESR1 *encodes estrogen receptor alpha which regulates estrogen signaling upon estrogen binding. Abnormal estrogen signaling leads to the development of a variety of diseases, such as cancer, metabolic and cardiovascular disease, neurodegeneration, inflammation, and osteoporosis (Jia et al., 2015). *NCOA3 *encodes nuclear receptor coactivator 3, a member of the nuclear receptor co-activator family known to be overexpressed in breast cancer and essentially involved in estrogen-mediated cancer cell proliferation (Wagner et al., 2013). Overexpression of *NCOA3* promotes breast cancer chemoresistance to tamoxifen (Burwinkel et al., 2005) and paclitaxel (Ao et al., 2016). *NCOA3* also drives the formation of cancer stem-like cells and supports tumor outgrowth in breast cancer models (Rohira et al., 2017). Moreover, *NCOA3* is a selective co-activator of ERα-mediated transactivation of PLAC1, novel cancer-associated placental in MCF-7 breast cancer cells (Wagner et al., 2013). *RPS6KB1* encodes ribosomal protein S6 kinase B1 which plays a key role in regulating protein translation and progression of hepatocellular carcinoma (Li et al., 2012), prostate cancer (Cai et al., 2015) and small cell lung cancer (Chen et al., 2017). S6K1 also activates ERα and promotes the proliferation of estrogen receptor-positive breast cancer cells (Holz, 2012). Taken together, those three genes regulate estrogen signaling in breast cancer and could be evaluated for further studies of marker and target genes of nobiletin in breast cancer stem cells.

Previous studies showed the role of nobiletin in estrogen, Wnt/ß-catenin and mTOR signaling. In estrogen signaling, nobiletin prevents bone loss due induced by estrogen deficiency in rats (Harada et al., 2011; Matsumoto et al., 2018) and inhibits lower cytotoxicity on MCF-7 estrogen receptor-positive breast cancer cells than on SKBR3 HER2 positive and MDA-MB 468 triple-negative breast cancer cells (Chen et al., 2014). Moreover, treatment of nobiletin in lower dose decreases activity and expression of aromatase on MCF-7 cells (Rahideh et al., 2017). In Wnt/ß-catenin signaling, nobiletin inhibits its signaling pathway in hypoxia stimulated Caki-1 and 786-O renal cell carcinoma (Liu et al., 2019), and inhibits invasion via inhibition of AKT/GSK3ß/ß-catenin pathway in glioblastoma cells (Zhang et al., 2017). Nobiletin shows inhibition of mTOR signaling on MDA-MB-468 triple-negative breast cancer cells (Chen et al., 2014). On the mTOR signaling pathway, nobiletin also protects cadmium-induced neurotoxicity induced by cadmium (Qu et al., 2018) and increases the sensitivity of colorectal cancer to oxiplatin (Li et al., 2019). Accordingly, those studies support the present study and enhance the development potential of nobiletin as CSCs-drugs by targeting estrogen, Wnt/ß-catenin and mTOR signaling. 

This present study showed that nobiletin target estrogen signaling and Wnt/ß-catenin signaling. Protein interaction networks showed three hub genes regulates estrogen signaling. A previous study also showed functional interaction between estrogen and Wnt/ß-catenin signaling (Kouzmenko et al., 2004). Estradiol not only stimulates the estrogen signaling pathway but also increases the cancer stem cell (CSC) population in estrogen receptor-positive breast cancer cells (Kurebayashi et al., 2017). Treatment with hormone antagonist in estrogen receptor-positive breast cancer cells may repress their estrogen receptors and be resistant to hormone therapy (Simoes et al., 2015). However, a recent study showed that tamoxifen-resistant cells exhibit increased stemness properties via activation of Wnt/ß-catenin signaling (Leung et al., 2017). The interaction of CXC chemokine receptor type 4 (CXCR4) with its ligand CXC motif ligand 12 (CXCL12) plays important roles in maintaining CSCs properties in tamoxifen-resistant breast cancer cells (Dubrovska et al., 2012), nasopharyngeal CSCs (Tian et al., 2017), esophageal CSCs (Wang et al., 2017b), and stimulates the angiogenesis in vascular endothelial cells through upregulation of the MAPK/ERK and PI3K/AKT and Wnt/β-catenin pathways. (Song et al., 2018). A study showed that nobiletin decreases the expression of *CXCR4 *in breast cancer cells (Baek et al., 2012). Accordingly, nobiletin is potential to target CSCs by inhibiting estrogen and Wnt/ß-catenin signaling. 

This present study has several limitations, including the mRNA data used for the PPI network. This might give different results because the expression of mRNA is not always correlated to the protein level. This study is also using bioinformatics approaches, therefore further in vitro and in vivo studies are needed to validate the results as well as to explore the full therapeutic potential of nobiletin on CSCs.

In conclusion, we found that tamoxifen, triciribine phosphate and 4-ipomeanol are standard drugs with the highest score of Pearson correlation coefficient to nobiletin. Moreover, many genes involved in protein binding, transcriptional and translational activity. Importantly, pathway enrichment analysis revealed breast cancer regulation of estrogen signaling and Wnt/ß-catenin by nobiletin. In addition, three hub genes, i.e. *ESR1*, *NCOA3*, and *RPS6KB1* and one significant module were filtered out and selected from the PPI network. Taken together, using a bioinformatics approach, we showed that nobiletin might serve as a lead compound for the development of cancer stem cells-targeted drugs by targeting targets estrogen and Wnt/ß-catenin signaling. 

## Availability of material

The datasets analysed during the present study are online available in the public database.
